# Sex Differences in Cardiovascular Mortality in Diabetics and Nondiabetic Subjects: A Population-Based Study (Italy)

**DOI:** 10.1155/2015/914057

**Published:** 2015-03-22

**Authors:** Paola Ballotari, Sofia Chiatamone Ranieri, Ferdinando Luberto, Stefania Caroli, Marina Greci, Paolo Giorgi Rossi, Valeria Manicardi

**Affiliations:** ^1^Servizio Interaziendale di Epidemiologia, Azienda Unità Sanitaria Locale, Via Amendola 2, 42122 Reggio Emilia, Italy; ^2^IRCCS Arcispedale Santa Maria Nuova, Viale Umberto I 50, 42123 Reggio Emilia, Italy; ^3^Laboratorio Analisi Chimico Cliniche ed Endocrinologia, IRCCS Arcispedale Santa Maria Nuova, Viale Risorgimento 80, 42123 Reggio Emilia, Italy; ^4^Dipartimento Cure Primarie, Azienda Unità Sanitaria Locale, Via Amendola 2, 42122 Reggio Emilia, Italy; ^5^Dipartimento di Medicina Interna, Ospedale di Montecchio, Azienda Unità Sanitaria Locale, Via Barilla 16, 42027 Montecchio, Italy

## Abstract

The objective of this study is to assess the impact of diabetes on cardiovascular mortality, focusing on sex differences. The inhabitants of Reggio Emilia province on December 31, 2009, aged 20–84 were followed up for three years for mortality. The exposure was determined using Reggio Emilia diabetes register. The age-adjusted death rates were estimated as well as the incidence rate ratios using Poisson regression model. Interaction terms for diabetes and sex were tested by the Wald test. People with diabetes had an excess of mortality, compared with nondiabetic subjects (all cause: IRR = 1.68; 95%CI 1.60–1.78; CVD: IRR = 1.61; 95%CI 1.47–1.76; AMI: IRR = 1.59; 95%CI 1.27–1.99; renal causes: IRR = 1.71; 95%CI 1.22–2.38). The impact of diabetes is greater in females than males for all causes (*P* = 0.0321) and for CVD, IMA, and renal causes. Further studies are needed to investigate whether the difference in cardiovascular risk profile or in the quality of care delivered justifies the higher excess of mortality in females with diabetes compared to males.

## 1. Introduction

Diabetes is now one of the most common noncommunicable diseases globally. The International Diabetes Federation (IDF) most recent estimates indicate that 8.3% of adults—382 million people—have diabetes. Further, the number of people with the disease is expected to rise beyond 592 million in less than 25 years. Yet with 175 million cases currently undiagnosed, a vast number of people who are unaware that they have diabetes are progressing towards complications [1]. In Italy, the overall prevalence in 2011 was about 5%; that is, 1,383,000 men and 1,556,000 women have diabetes [2].

Diabetes and its complications are major causes of early death in most countries. In Europe, one in 10 deaths in adults can be attributed to diabetes, that is 619,000 in 2013 [1]. Cardiovascular disease (CVD), the first cause of death in many industrialized countries, is responsible for a large part of the excess mortality observed among people with diabetes [3]. Indeed, individuals with diabetes have an increased risk of all-cause mortality and morbidity related to CVD compared with individuals without diabetes [3–9].

Nevertheless, the effect of diabetes on CVD seems to be different for males and females [10–17]. In fact, despite the fact that in many industrialized countries women have lower mortality rates than men, when we look at people with diabetes, the advantage for women is reduced or even absent [18, 19]. Estimates of CVD mortality in men with diabetes have varied from 1 to 3 times the rate in men free of the disease, whereas estimates in women with diabetes have ranged from 2 to 5 times the rate in women without diabetes [20–23]. The variation in relative risk estimates of cardiovascular disease makes it difficult to evaluate the strength of diabetes as a risk factor for either sex.

The objective of this study is therefore to assess the impact of diabetes on cardiovascular mortality, focusing on sex differences.

## 2. Methods

### 2.1. Setting and Study Population

This study is a retrospective cohort including the inhabitants of Reggio Emilia province (northern Italy) on December 31, 2009, aged 20–84.

To identify people with diabetes (i.e., exposed group) we used the Reggio Emilia diabetes register (accessed on May 21, 2014). The methods applied to develop our disease register have been described elsewhere [24]. In brief, the register was created by deterministic linkage of six routinely collected data sources through a definite algorithm able to ascertain cases and to distinguish type of diabetes and model of care. The sources are hospital discharge, drug dispensation, HbA1c values from biochemistry laboratory, disease-specific exemption, diabetes outpatient clinics, and mortality databases. Women with gestational diabetes or women receiving treatment for polycystic ovarian syndrome were excluded.

### 2.2. Follow-Up, Outcome, and Covariates

Cohort was followed up for three years (2010–2012). Vital status (alive or dead) information was retrieved from civil register. The subjects who emigrated were treated as censored at the time of emigration.

The outcome of interest was mortality attributable to all causes (ICD-10 A00-T98), cardiovascular disease (CVD) (ICD-110 I00-I99), acute myocardial infarction (AMI) (ICD-10 I21-I23), diabetes (ICD-10 E10-E14), and renal diseases (ICD-10 N00-N39). The causes of death were ascertained using Reggio Emilia mortality register, which contains all resident deaths by year of death, with cause of death coded using* International Classification of Diseases*, tenth revision (ICD-10). Sex and age were considered covariates in the analysis. As a proxy of disease severity, the subjects with diabetes were classified based on treatment: diet only, oral antidiabetic drugs, or insulin. Subjects who were prescribed both insulin and oral antidiabetic drugs were assigned to “insulin treatment” [25].

### 2.3. Statistical Methods

Characteristics of the study population are presented as median and proportions and stratified by sex and diabetes status. Person-time at risk was calculated from January 1, 2010, to date of death or date of emigration or December 31, 2012.

We calculated proportional mortality by age and diabetes status for principal groups of cause of death.

Then we estimated age-adjusted death rates (AADR) per 100000 with 95% confidence intervals (95% CI), by sex and diabetes status using Italian population on December 31, 2009, as reference for standardization [26]. At the same time, we calculated incidence rate ratios (IRR) and 95% confidence intervals (95% CI) using multivariate Poisson regression model. The individuals without diabetes were used as the reference group, the age as continuous variable, and the sex as covariate. Interaction terms for diabetes and sex were tested by the Wald test.

Further, we estimated incidence rate ratios (IRR) and 95% confidence intervals (95% CI) and risk difference (per 100000) within age category, for all causes, CVD, and AMI and renal causes, and we graphed the age-specific death rates stratified by sex and diabetes status.

Analyses were performed using the STATA statistical package, version 11.0.

### 2.4. Ethical Approval

This is an observational study and data were collected retrospectively. The Local Health Authority of Reggio Emilia was responsible for collecting and processing these sets of data. The study was commissioned by the Local Health Authority. The Reggio Emilia diabetes registry was approved by provincial Ethic Committee in July 2014. According to Italian privacy law, no patient or relative's consent is required for large retrospective population-based studies.

## 3. Results

The study cohort consisted of 407,161 subjects (Table 1), 23,438 of whom were diabetic patients (i.e., exposed group) (5.8% of the population): 13074 males and 10364 females (prevalence 6.5% and 5.0%, resp.). Subjects without diabetes were younger and there was a higher percentage of foreigners. The percentage of lost to follow-up because of move was very low in both groups.

Over the three-year study period, 9,208 (2.3%) individuals died; the proportion of deaths was higher in people with diabetes than the unexposed population (8.7% and 1.9%, resp.). The risk was greater in males than females in both groups.

Finally, among people with diabetes, there were no differences by sex in terms of type of treatment (*P* = 0.120).

The distribution of causes was similar for the two populations (Table 2), with the exception of death for endocrine, nutritional, and metabolic causes (which includes diabetes) (E00-E90), where the percentage was 10.3% for males and 11.4% for females with diabetes, compared to 0.7% and 0.9% for males and females without diabetes, respectively. The pattern of mortality by sex was similar in the two subgroups, except for the digestive and renal causes. In females, the proportion of deaths for CVD was 33.2%, with a slight difference between diabetics and nondiabetics subjects (34.4% and 32.9% resp.); in males the percentage of deaths for CVD causes was lower (28.5%) and similar in the two groups.

Diabetic subjects showed an increased risk of all-cause mortality compared to nondiabetics of dying for all causes (Table 3). The excess of risk was found in all categories of causes analyzed in our study.

The analysis by sex indicated that the excess of risk was more evident in diabetic females than diabetic males compared to their nondiabetic counterparts (IRR 1.77; 95% CI 1.64–1.92; IRR 1.63; 95% CI 1.52–1.73, resp.). The effect modification of sex on the association between diabetes and death was statistically significant (Wald test for interaction, *P* = 0.0321).

Looking at cardiovascular mortality, we observed a similar pattern: an excess of risk in people with diabetes, found in both sexes, greater in females than males (males: IRR 1.56; 95% CI 1.38–1.76; females: IRR 1.69; 95% CI 1.47–1.93; Wald test for interaction, *P* = 0.1266).

Among the CVD causes, we observed that for AMI the excess mortality for females with diabetes was more pronounced (males: IRR 1.48; 95% CI 1.10–1.99; females: 1.81; 95% CI 1.27–2.59; Wald test for interaction, *P* = 0.1063).

In the group of renal causes of death, the excess of mortality in the diabetic population was again more evident in females than males (males: IRR 1.37; 95% CI 0.88–2.14; females: 2.37; 95% CI 1.43–3.91; Wald test for interaction, *P* = 0.1466). This group of causes includes those related to kidney dysfunctions, such as glomerular diseases, renal tubulointerstitial diseases, acute kidney failure, chronic kidney disease, and other disorders of the kidney and urethra. In this group, the deaths caused by renal failures were 70% of the total in the diabetic population, while the percentage decreased to 59% in nondiabetic population. In both subgroups, the remaining deaths were almost entirely ascribed to “other diseases of urinary system” block.

Comparing number of deaths by cause among diabetic and nondiabetic individuals, we observed 214 deaths caused by diabetes in the former subgroup and 31 in the latter (this subgroup included people with diabetes diagnosed after 2009), corresponding to a cause-specific age-adjusted death rate of 129.3 and 3.1 per 100000 p/y, respectively. The presence of the diabetes-specific cause makes it difficult to compare the other causes of mortality between the two populations. In fact, this cause of death subtracts cases to other causes and in particular to cardiovascular and renal causes, because often the final cause was attributable to one of these two categories.

Analysis of incidence rate ratios by age class suggests that the impact of diabetes decreases with increasing age (Table 4). The effect can only be observed in all-cause mortality and CVD as a whole, because the absolute AMI and renal causes risk of death are too small in younger ages. Nevertheless, the risk difference increased with age, reaching 26.3 per 1000 p/y in males aged 75–84 for all causes and 21.1 in females, while in 20–34-year-old class the difference was 1.6 per 1000 p/y in males and 1.3 in females. In case of CVD, the risk difference in the oldest age class reached 7 per 1000 p/y, in both sexes.

**Figure 1:**
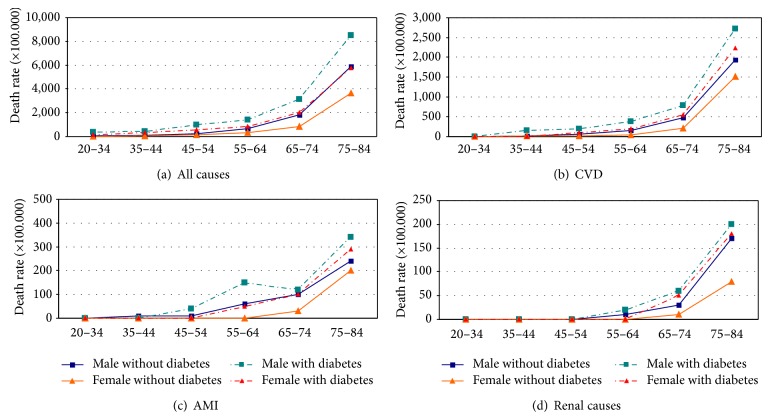
Age-specific death rates by sex and diabetes status: (a) all cause of death; (b) CVD; (c) AMI; (d) renal causes. The curves with dash indicate diabetic patients and the curve with squares indicate males and the curve with triangles indicate females.

Comparison among age-specific death rates by sex and diabetes status (Figure 1) indicated that males with diabetes have the highest rates. However, females with diabetes have higher rates than males without diabetes mainly in the younger age groups, while females without diabetes have very low death rates until the age of 64.

## 4. Discussion

Our study found an excess of mortality associated with diabetes in both sexes, for all causes and for all groups of causes analyzed. However, the excess in the ratios was limited compared to findings of other studies [9, 27–29]. It must be emphasized that our study was population-based and data on exposure were retrieved from a register built using six different sources, assuring sensitivity and specificity [24]. This study design includes a wider denominator of exposed people compared to studies where the cohort is hospital or treatment based.

Focusing on CVD causes, the risk of death for diabetics is 61% higher than that for nondiabetics subjects (95% CI: 1.47–1.76), with no differences between the two subcategories, AMI and “other CVD causes”.

Considering all causes of death, our study found evidence of greater impact of diabetes on females than males, despite the severity of disease seeming to be similar in the two groups, in agreement with a recent population-based retrospective cohort study [18]. When we analyzed CVD, IMA, and renal causes, the different effect of diabetes by sex was also present, although the power of the study does not permit ruling out the possibility that the difference was due to random fluctuations.

The reason why diabetes determines a greater excess of all-cause mortality in females than males is not completely understood, especially for CVD causes [30].

One explanation is that type-2 diabetes mellitus (T2DM) may reduce the advantage of females in the prevalence of cardiovascular disease by fading the vascular protective effects given by estrogens [21, 31–33]. Many authors have suggested that the CVD risk factors have a stronger impact on females than males [14, 15, 32–34]. Compared to males, females with diabetes have a worse cardiovascular profile, which could explain their higher cardiovascular mortality, mainly at age <60. Females with diabetes have higher prevalent abdominal obesity [34, 35], increasing the risk of hypertension [19, 36], a worse lipid profile, since the onset of diabetes (low levels of HDL cholesterol [HDL-C], small particle size of LDL cholesterol [LDL-C], and high levels of triglycerides) [35, 37–39], and a more marked endothelial dysfunction than males with diabetes [40–45], a greater degree of fibrinolysis/thrombosis compared to males [46, 47], and also an increased prevalence of hypoglycemic events compared to that of male diabetic patients [48]. These phenomena might explain the increased incidence of cardiovascular events and mortality among female patients [49].

Besides innate differences in sex physiology, disparities between sexes in the treatment of major cardiovascular risk factors also exist [35, 50, 51]. These can be attributed to an underestimation of patient risk and a less aggressive approach (i.e., prescription of lower doses) and poorer compliance of females [52–55]. Nevertheless, two Italian studies did not find any relevant differences between females and males in terms of the quality of diabetes care [35, 56]. In one of these recent large cross-sectional studies, women were less likely to reach the recommended targets despite receiving the same treatment for lipid control and hypertension and they were more likely to be overtreated with insulin. Women still showed a lower likelihood of being monitored for diabetes complications, particularly foot and eye complications. As for intermediate outcomes, the proportion of individuals reaching the targets of HbA1c, LDL cholesterol, and BMI values was systematically lower for women than men. The only result that went in the opposite direction was that, among diabetic patients with high LDL cholesterol, a higher proportion of women were not treated with lipid-lowering therapy [35].

In our study we also investigated mortality caused by renal diseases and in particular codes N00-N39, that is, glomerular diseases, renal tubulointerstitial diseases, acute kidney failure and chronic kidney disease, urolithiasis, other disorders of kidney and urethra, and other diseases of the urinary system, given the close interconnection between renal and cardiovascular disease.

For this group as well, we found risk excess in diabetic population and the excess was stronger in females than males. This excess in females is closely linked to CVD mortality and could partially explain its increase [57–59]. There are few sets of data on the role of gender on microvascular complications and increasing mortality related to them [60]. While females in the general population have less renal disease, this advantage is less evident in diabetic nephropathy than nondiabetic kidney diseases [61–63]. The contribution of sex to diabetic renal disease is still unclear. Although some studies indicate that females progress at a faster rate [64], others studies indicate the opposite [65–67]. Some studies suggest that male sex remains a risk factor for the development of micro- and macroalbuminuria as well as the progression of an established diabetic nephropathy [68]. However, the prevalence of a reduced glomerular filtration rate estimated in females was higher than that in males [69]. This nonalbuminuric renal impairment phenotype is associated with higher incidence of CVD, particularly in the coronary district [70].

The differences in therapy effectiveness in females as well as the existence of different disease pathways in the kidney and cardiovascular disease have led some authors to suggest the need to develop gender-specific therapeutic strategies to prevent renal dysfunction and reduce associated morbidity and mortality in females [71].

Nevertheless, it is important to note that even if the incidence rate ratios declined with age, the risk difference increased in the older groups, where the number of deaths is much higher. In other words, in a hypothetical population free of diabetes disease, in age class 75–84 years, 26 deaths for every 1,000 males and 21 deaths for 1,000 females still alive would be avoided, while the savings would be 1.6 for 1,000 males and 1.3 for 1,000 females in the age class 20–34 years.

The particularly high excess of risk in younger ages is mainly due to low mortality in nondiabetic group, and the phenomenon is more pronounced in females. Our results agree with other studies [69, 70].

## 5. Strengths and Limitations

This is a population-based cohort study using data from a province-wide diabetes register for exposure identification and from mortality register for case detection, thereby reducing misclassification bias. Moreover, while there have been several studies on all causes and CVD mortality among people with diabetes, this is one of the few studies exploring the effect of diabetes on renal causes mortality. Finally, our study focused on the greater impact of diabetes on female mortality, exploring possible hypotheses for this phenomenon.

However, this study considered only age as confounder; other possible confounders, such as socioeconomic characteristics, behavioral risk factors (i.e., BMI, smoking), and clinical information other than treatment, such as duration of disease and micro- and macrovascular diabetes complications, were not considered.

Finally, the presence of diabetes as cause of death makes it difficult to compare the cause-specific mortality between the population with and without diabetes, in particular for CVD and renal diseases.

## 6. Conclusions

Diabetes determines a 68% excess in mortality rate. The relative risk for diabetic patients versus nondiabetic population is particularly relevant in young and middle-aged subjects, where diabetes status contributes to occurrence of deaths that are unexpected in nondiabetic population. Furthermore, diabetes has a greater impact on females than males, reducing the advantage of females in all-cause mortality as well as CVD, in particular AMI, and renal mortality observed in the population without diabetes.

Further studies are needed to determine whether the difference in cardiovascular risk profile or the quality of care delivered justifies the higher excess of mortality in females with diabetes than males.

## Figures and Tables

**Table 1:** Characteristics of the study cohort by diabetes status and sex.

Characteristics	No diabetes		Diabetes		Total	
	Males	Females	Males	Females	Males	Females
Population 20–84 years	187886	195837	13074	10364	200960	206201
Foreigners^*^: *N* (%)	25749 (13.7)	28104 (14.3)	950 (7.3)	862 (8.3)	26699 (13.3)	28966 (14.0)
Age (years): median (IQR)	44 (34–59)	47 (35–62)	66 (57–74)	69 (60–76)	46 (35–61)	48 (36–63)
Dead: *N* (%)	4090 (2.2)	3090 (1.6)	1240 (9.5)	788 (7.6)	5330 (2.6)	3878 (1.9)
Emigrated: *N* (%)	417 (0.2)	319 (0.2)	16 (0.1)	12 (0.1)	433 (0.2)	331 (0.2)
Person-years	558521	583162	37234	29842	595755	613004
Diabetes treatment regimen						
Diet only			3213 (24.6)	2626 (25.3)		
Oral drugs			6760 (51.7)	5219 (50.4)		
Insulin			3101 (23.7)	2519 (24.3)		

^*^Based on the country of birth.

**Table 2:** Proportional mortality by diabetes status and sex.

Causes: *N* (%)	No diabetes		Diabetes		Total	
	Males	Females	Males	Females	Males	Females
Infectious and parasitic diseases (A00–B99)	97 (2.4)	72 (2.3)	39 (3.1)	30 (3.8)	136 (2.6)	102 (2.6)
Neoplasms (C00–D48)	1680 (41.1)	1176 (38.1)	426 (34.4)	239 (30.3)	2106 (39.5)	1415 (36.5)
Endocrine, nutritional, and metabolic diseases (E00–E90)	30 (0.7)	29 (0.9)	128 (10.3)	90 (11.4)	158 (3.0)	119 (3.0)
Mental and behavioral disorders (F00–F99)	61 (1.5)	87 (2.8)	13 (1.0)	7 (0.9)	74 (1.4)	94 (2.4)
Diseases of the nervous system (G00–G99)	146 (3.6)	132 (4.3)	19 (1.5)	23 (2.9)	165 (3.1)	155 (4.0)
Diseases of the circulatory system (I00–I99)	1161 (28.4)	1017 (32.9)	357 (28.8)	271 (34.4)	1518 (28.5)	1288 (33.2)
Diseases of the respiratory system (J00–J99)	335 (8.2)	205 (6.6)	102 (8.2)	38 (4.8)	437 (8.2)	243 (6.3)
Diseases of the digestive system (K00–K93)	154 (3.8)	130 (4.2)	67 (5.4)	36 (4.6)	221 (4.1)	166 (4.3)
Renal causes (N00–N99)	87 (2.1)	59 (1.9)	25 (2.0)	21 (2.7)	112 (2.1)	80 (2.1)
Injury, poisoning, and other certain consequences of external causes (S00–T98)	244 (6.6)	110 (3.6)	44 (3.5)	17 (2.2)	288 (5.4)	127 (3.3)
Unknown	46 (1.1)	22 (0.7)	13 (1.0)	5 (0.6)	59 (1.1)	27 (0.7)
Other^*^	49 (1.2)	51 (1.7)	7 (0.6)	11 (1.4)	56 (1.1)	62 (1.6)
Total (A00–T98)	**4090**	**3090**	**1240**	**788**	**5330**	**3878**

^*^Others include cases classified in the following chapters: III, Diseases of the blood and blood-forming organs and certain disorders involving the immune mechanism (D50–D89) (*N* = 20); VII, Diseases of the eye and adnexa (H00–H59) (*N* = 1); VIII, Diseases of the ear and mastoid process (H60–H95) (*N* = 1); XII, Diseases of the skin and subcutaneous tissue (L00–L99) (*N* = 14); XIII, Diseases of the musculoskeletal system and connective tissue (M00–M99) (*N* = 34); XVII, Congenital malformations, deformations and chromosomal abnormalities (Q00–Q99) (*N* = 11); XVIII, Symptoms, signs and abnormal clinical and laboratory findings, not elsewhere classified (R00–R99) (*N* = 37).

**Table 3:** *N* of dead, age-adjusted death rates (AADR) per 100.000 person/years and incidence rate ratios (IRR) with 95% confidence intervals (95% CI) by sex and diabetes status, for cause of death.

Causes	Males					Females					Total				
	No diabetes		With diabetes			No diabetes		With diabetes			No diabetes		With diabetes		
	*N*	AADR (95% CI)	*N*	AADR (95% CI)	IRR (95% CI)	*N*	AADR (95% CI)	*N*	AADR (95% CI)	IRR (95% CI)	*N*	AADR (95% CI)	*N*	AADR (95% CI)	IRR (95% CI)
All causes (A00–T98)	4090	869.5 (843.3–895.6)	1240	1662.9 (1432.5–1893.2)	1.63 (1.52–1.73)	3090	600.5 (579.6–621.4)	788	1197.0 (1077.5–1316.4)	1.77 (1.64–1.92)	7180	730.9 (714.2–747.5)	2028	1422.7 (1295.3–1550.2)	1.68 (1.60–1.78)
CVD (I00–I99)	1161	249.8 (235.5–264.0)	357	432.5 (373.8–491.2)	1.56 (1.38–1.76)	1017	200.0 (187.8–212.2)	271	360.3 (315.0–405.5)	1.69 (1.47–1.93)	2178	224.1 (214.8–233.5)	628	395.3 (358.5–432.0)	1.61 (1.47–1.76)
*AMI* *(I21–I23) *	*221 *	*46.4 (40.2–52.5) *	*57 *	*70.9 (50.6–91.1) *	*1.48 (1.10–1.99) *	*137 *	*26.9 (22.4–31.4) *	*39 *	*51.6 (34.9–68.3) *	*1.81 (1.27–2.59) *	*358 *	*36.3 (32.6–40.1) *	*96 *	*60.9 (47.9–74.0) *	*1.59 (1.27–1.99) *
*Other CVD *	*950 *	*203.4 (190.5–216.3) *	*300 *	*361.6 (306.5–416.8) *	*1.58 (1.39–1.80) *	*880 *	*173.1 (161.7–184.5) *	*232 *	*308.6 (266.5–350.8) *	*1.67 (1.44–1.93) *	*1820 *	*187.8 (179.2–196.4) *	*532 *	*334.3 (299.9–368.8) *	*1.62 (1.47–1.78) *
Diabetes (E10–E14)	18	3.8 (2.0–5.6)	125	139.9 (114.2–165.6)	37.22 (22.61–61.30)	13	2.5 (1.1–3.9)	89	119.4 (90.9–147.9)	44.79 (24.98–80.31)	31	3.1 (2.0–4.2)	214	129.3 (110.1–148.6)	40.46 (27.68–59.14)
Renal causes (N00–N39)	87	19.0 (15.0–23.0)	25	26.1 (15.8–36.4)	1.37 (0.88–2.14)	59	11.5 (8.6–14.4)	21	26.0 (14.8–37.1)	2.37 (1.43–3.91)	146	15.1 (12.7–17.6)	46	26.0 (18.4–33.6)	1.71 (1.22–2.38)
Other causes	2824	596.9 (575.1–618.7)	733	1064.4 (842.9–1285.8)	1.44 (1.32–1.56)	2001	386.5 (369.7–403.4)	407	691.3 (584.5–798.1)	1.51 (1.35–1.68)	4825	488.5 (474.8–502.2)	1140	872.1 (751.5–992.7)	1.46 (1.37–1.56)

CVD = cardiovascular disease; AMI = acute myocardial infarction; AADR = age-adjusted death rate, using Italian population at 31.12.2009, stratified by sex.

IRR = calculated using Poisson model, adjusted for age, and sex. People without diabetes were used as reference.

**Table 4:** Incidence rate ratios (IRR) with 95% confidence intervals (95% CI) and risk difference (RD) per 1000 p/y within age categories by sex, diabetics versus nondiabetic subjects, for all causes, CVD, AMI, and renal causes.

Age	All				CVD				AMI				Renal causes			
	Males		Females		Males		Females		Males		Females		Males		Females	
	IRR	RD	IRR	RD	IRR	RD	IRR	RD	IRR	RD	IRR	RD	IRR	RD	IRR	RD
20–34	8.03 (0.95–30.23)	3.1	7.02 (0.17–42.14)	1.4	—	—	—	—	—	—	—	—	—	—	—	—
35–44	3.68 (1.56–7.43)	2.9	5.60 (1.49–14.93)	2.7	6.60 (1.29–21.09)	1.3	—	—	—	—	—	—	—	—	—	—
45–54	3.86 (2.80–5.22)	7.1	3.83 (2.13–6.42)	4.0	4.14 (1.87–8.29)	1.4	6.73 (1.26–23.51)	0.8	2.65 (0.29–11.40)	0.3	—	—	—	—	—	—
55–64	2.18 (1.78–2.64)	7.5	2.51 (1.84–3.35)	5.0	2.51 (1.68–3.66)	2.3	4.50 (2.20–8.65)	1.6	2.29 (1.66–4.21)	0.9	9.88 (1.45–58.39)	0.5	2.67 (0.26–14.92)	0.1	—	—
65–74	1.72 (1.52–1.93)	13.1	2.32 (1.96–2.73)	11.3	1.62 (1.27–2.06)	3.0	2.68 (1.91–3.69)	3.4	1.13 (0.58–2.04)	0.2	3.26 (1.38–7.17)	0.7	1.88 (0.66–4.71)	0.3	4.48 (1.15–15.54)	0.4
75–84	1.45 (1.32–1.58)	26.3	1.58 (1.43–1.74)	21.1	1.40 (1.20–1.64)	7.8	1.47 (1.26–1.72)	7.2	1.45 (0.90–2.26)	1	1.45 (0.91–2.25)	0.9	1.17 (0.63–2.05)	0.3	2.25 (4.10–6.07)	1
